# Application of Motif-Based Tools on Evolutionary Analysis of Multipartite Single-Stranded DNA Viruses

**DOI:** 10.1371/journal.pone.0071565

**Published:** 2013-08-06

**Authors:** Hsiang-Iu Wang, Chih-Hung Chang, Po-Heng Lin, Hui-Chuan Fu, ChuanYi Tang, Hsin-Hung Yeh

**Affiliations:** 1 Department of Computer Science, National Tsing Hua University, Hsinchu, Taiwan; 2 Department of Computer Science and Information Engineering, Providence University, Taichung City, Taiwan; 3 Department of Plant Pathology and Microbiology, National Taiwan University, Taipei, Taiwan; 4 Research Center for Plant Medicine, National Taiwan University, Taipei, Taiwan; University of Hong Kong, China

## Abstract

Multipartite viruses contain more than one distinctive genome component, and the origin of multipartite viruses has been suggested to evolve from a non-segmented wild-type virus. To explore whether recombination also plays a role in the evolution of the genomes of multipartite viruses, we developed a systematic approach that employs motif-finding tools to detect conserved motifs from divergent genomic regions and applies statistical approaches to select high-confidence motifs. The information that this approach provides helps us understand the evolution of viruses. In this study, we compared our motif-based strategy with current alignment-based recombination-detecting methods and applied our methods to the analysis of multipartite single-stranded plant DNA viruses, including bipartite begomoviruses, Banana bunchy top virus (BBTV) (consisting of 6 genome components) and Faba bean necrotic yellows virus (FBNYV) (consisting of 8 genome components). Our analysis revealed that recombination occurred between genome components in some begomoviruses, BBTV and FBNYV. Our data also show that several unusual recombination events have contributed to the evolution of BBTV genome components. We believe that similar approaches can be applied to resolve the evolutionary history of other viruses.

## Introduction

Multipartite viruses contain more than one genome component (or segment), and for a multipartite virus to initiate a successful infection, all of the genome components must infect the same host cell and simultaneously replicate within the cell. Because the genome components of a multipartite virus can utilize the same protein(s) for replication and encapsidation, conserved sequences can be observed within these genome components. Aside from the small conserved region, the nucleotide sequences of the remaining genome components are usually quite different from each other. It has been suggested that multipartite viruses evolved from a non-segmented wild-type virus, although different factors favoring the generation of segmented genomes have been proposed [1], [2]. Recombination events have been found between genome components in a variety of multipartite viruses [3], [4], [5], [6], [7], [8], [9].

Viruses evolved to have a fast replication cycle, and some studies have indicated that recombination events occur frequently [10], [11], [12], [13], [14]. The rearranged genome could cause non-functional proteins to be produced; however, these recombined genome components might be maintained through complementation supported by other genome components or the original un-recombined genome component. Collectively, if recombination accumulated during the long evolution process in the small genome of a virus, then the sequence of the recombined genome could be shuffled, and we would not be able to align it well.

Phylogeny-based methods are the most commonly used methods for detecting recombination from the standpoint of evolutionary histories [15], [16]. These methods are usually designed to slide a window along the aligned sequences and to monitor the phylogenetic variation (e.g., the tree topology), to locate the recombination breakpoint. Formally, phylogeny-based methods require a multiple sequence alignment as the first step. Phylogeny-based methods allow a comparison of the gene sequences of different genomes. However, it is difficult to deduce the evolutionary relationships of genomes that cannot be aligned [17].

An alternative to alignment-based evolutionary analysis, the rearrangement distance algorithm, was presented by Sankoff [18] for the analysis of the evolutionary relationships between genomes. The first step in this method is the identification of homologous genes or common regions shared between the genomes of progeny, followed by the use of these genes or segments as markers [18], [19]. Later, the evolutionary distance can be calculated by the recombinational rearrangement steps that are necessary to convert the order of the markers in one genome to the order in another. The accuracy of these methods largely relies on the resolution of the markers between the genomes of the progeny.

Motifs are sequence patterns that recur in different genome regions; these patterns could have some biological significance, such as being protein binding sites of regulatory proteins or being associated with the structural motifs of proteins. Several motif-finding tools have been developed that can detect small stretches of recurrent sequences [20]. Thus, we think that a motif-finding tool can be applied to detect possible sequence patterns that recur in different viral genome components of multipartite viruses; these patterns might have some biological significance [20]. Moreover, the recurring motifs can be applied to studies of evolution.

In this study, we employed a motif-finding method and computational simulation to detect recombination events for phylogenetic analysis. We applied our methods to the analysis of multipartite plant DNA viruses, including bipartite begomoviruses, Banana bunchy top virus (BBTV) and Faba bean necrotic yellows virus (FBNYV). Our analysis revealed that several unexpected recombination events contributed to the evolution of these viruses. We believe that similar approaches can be applied to resolve the evolutionary history of other viruses.

## Results

### Comparison of alignment- and motif-based recombination detection methods

**Figure 1:**
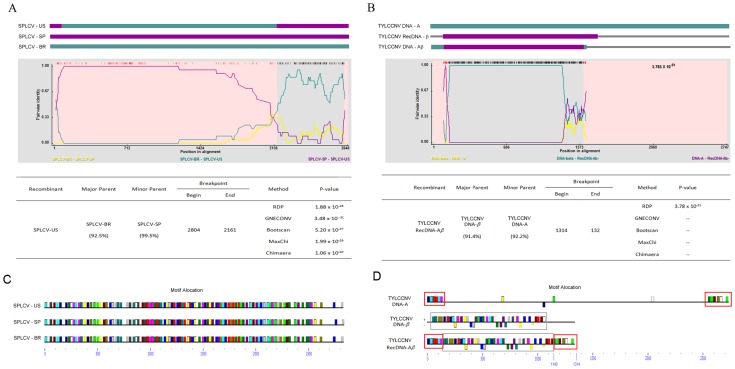
Recombination detection results of (A) SPLCV and (B) TYCCNV. The schematic representations based on alignments of Sweet potato leaf curl virus (SPLCV) isolates (A) and Tomato leaf curl China virus (TYCCNV) components (B) are shown at the top of the figure, which indicates recombination events detected by RDP4 [29]. Each sequence is represented by an open rectangle and colored differently from the other sequences. The details of the recombination breakpoint detected by RDP4 are shown. The motifs detected by MEME in the genome of SPLCV and TYCCNV are shown at (C) and (D), respectively, and the same motifs are in the same color. Identical motifs in the TYLCCNV genomes are indicated by open rectangles.

To compare the alignment and motif-based recombination detection methods, we utilized published data of geminivirus for our initial trial. We first selected a monopartite begomovirus, Sweet potato leaf curl virus (SPLCV), which infects sweet potato, for analysis [21]. Three isolates, SPLCV-BR (HQ393455), -SP (HQ393473) and -US (HQ393450), were selected for analysis (Table S1 A). The SPLCV-US isolate is a recombinant of the putative parents SPLCV-BR and SPLCV-SP [21]. We repeated an alignment-based recombination analysis by use of BOOTSCAN [22], CHIMAERA [23], GENECONV [24], MAXCHI [25], RDP [26], SISCAN [27] and 3SEQ [28] implemented in the program RDP4 (Version 4.16) [29] to detect recombination in these isolates, and similar results were obtained (Figure 1A).

For motif-based recombination detection methods, we selected the motif-finding tool *Multiple Em for Motif Elicitation* (MEME) (http://meme.nbcr.net/meme/) in our analysis [30], [31]. We used MEME to detect the common motifs between SPLCV-PR, -SP and -US (Figure 1C). MEME detected identical motif locations in each genome and was unable to detect any recombination event. This indicated that when analyzing genome sequences that are similar and well aligned, alignment-based methods are better than motif-based methods in detecting recombination events.

Next, we selected another monopartite begomovirus, Tomato leaf curl China virus (TYLCCNV), for analysis [32]. Satellite molecules, named DNA-*β*, associate with DNA-A of TYLCCNV, which is essential for the induction of symptoms. Additionally, recombinant DNA molecules (RecDNA-A*β*) between DNA-A and DNA-*β* of TYLCCNV have been reported [32]. We used the genome sequences of DNA-A (AJ319675), DNA-*β* (AJ421621) and RecDNA-A*β* (AJ781297) for analysis (Listed in Table S1A). Both alignment- and motif -based recombination methods were performed (Figure 1B and 1D). The motif allocation detected by MEME clearly indicated that the genome sequence of RecDNA-A*β* had recombined with sequence derived from DNA-*β* (130–1140 nt position) and with sequences derived from DNA-A at the beginning and the end of the genome alignment (1–134 and 1140–1335 nt positions) (Figure 1B). However, the alignment-based methods were not able to detect the recombination at position 1140–1314 (Figure 1D) because this region could not be aligned well. The comparative analysis revealed that the alignment-based recombination detection methods are suitable for analyzing recombination events when sequences share high sequence similarities and can be easily aligned, and the motif-based recombination detection methods are suitable for analyzing recombination events when sequences share fewer similarities or cannot easily be aligned.

### Identification of common motifs between components A and B of bipartite begomoviruses by MEME

We next aimed to determine whether recombination events happened between genome components of multipartite viruses that are considered to contain distinct component sequences that only share short stretches of conserved sequences. We first analyzed if recombination events happened between the A and B components of all bipartite begomovirus listed in the Virus Taxonomy of International Committee on Taxonomy of Viruses (ICTV), including those that have not been approved as species (Table S1) [23].

### Identification of high-confidence motifs between components A and B of bipartite begomoviruses by MEME

**Figure 2:**
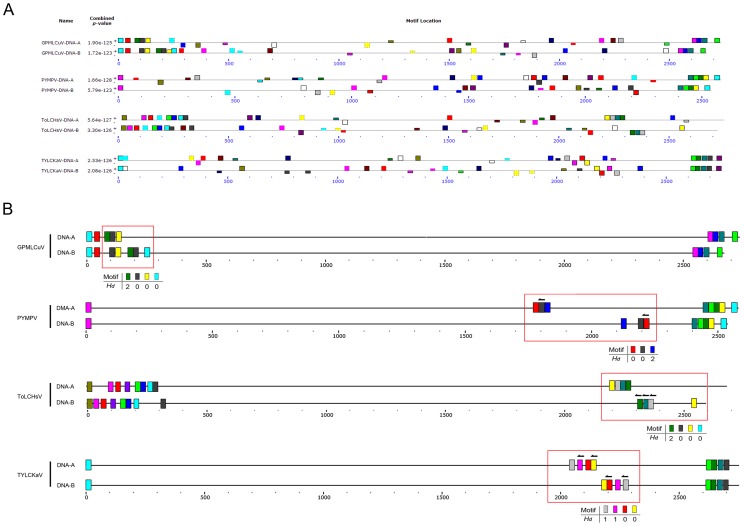
The motif distribution of begomovirus with genome rearrangement. (A) The distribution of motifs in the genomes of DNA-A and DNA-B components of Potato yellow mosaic Panama virus (PYMPV), *Gossypium punctatum* mild leaf curl virus (GPMLCuV), Tomato leaf curl Hsinchu virus (ToLCHsV) and Tomato yellow leaf curl Kanchanaburi virus (TYLCKaV), detected by MEME [31]. (B) Schematic representation of the locations of high-confidence motifs. The genome sequences are represented by gray lines. The colored rectangles on the genomes represent the identified motifs, and arrows indicate that the motif is reversed. The motifs belonging to the same set in the same genome are indicated in the same color. Potential recombinant regions are indicated by red open rectangles, and the *H_d_* of each motif set in the regions is listed.

**Figure 3:**
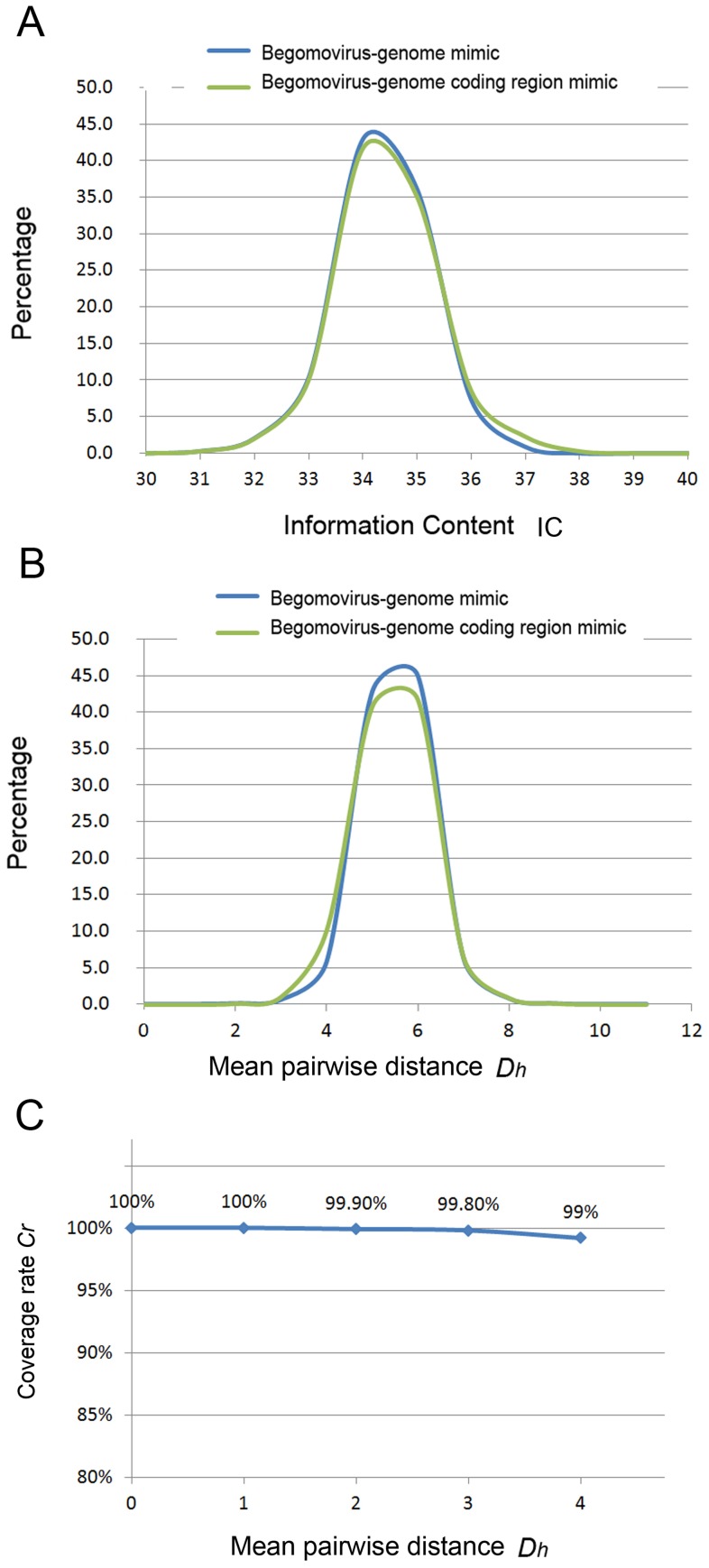
The evaluation of motifs in bipartite begomoviruses. (A) The *x*-axis represents the IC. The *y*-axis represents the percentage of motif sets with a certain IC. The green line and blue line represent the distribution of the percentage of the motif sets detected by the simulation of the begomovirus-genome coding region mimic sequence set and the begomovirus-genome random mimic sequence set, respectively. (B) The *x*-axis represents the mean pairwise distance (*D_h_*). The *y*-axis represents the percentage of motif sets with a certain *D_h_* value. The green line and blue line represent the distribution of the percentage of the motif sets detected by the simulation of the begomovirus-genome coding region mimic sequence set and the begomovirus-genome random mimic sequence set, respectively. (C) The percentage of motifs detected by MEME from motifs inserted in randomly generated sequences. The *D_h_* of each MEME-detected motif set is designated with the numbers 1 to 4. The *x*-axis represents the *D_h_* of artificial motifs that were generated and inserted randomly in begomovirus-genome mimic sequences The *y*-axis represents the coverage rate *C_r_* of detected motifs compared with the initially inserted artificial motifs.

In total, 86 bipartite begomoviruses were analyzed by MEME (Figure 2A and data not shown). To understand whether the motifs that were detected by MEME might have significance in the evolutionary history of the viruses, we first generated several sequence sets and subjected them to MEME analysis to identify the IC value for evaluation (Figure 3A). To identify the maximum IC value, we generated identical sets, each set including two identical sequences whose length was the average of the corresponding DNA-A and DNA-B genome components of bipartite begomoviruses. We also generated begomovirus-genome random mimic sequence sets by simulating the bipartite begomovirus genome. Each random sequence had an identical length and GC content corresponding to a specific component of the bipartite begomovirus genome. We applied random mimic sequence sets to identify an IC value, which was analyzed by MEME. To provide better control to reflect the associations of non-coding and coding sequence stretches of begomovirus, we also generated begomovirus-genome coding region mimic sequence sets. Each begomovirus-genome coding region mimic sequence set comprised two sequences, each generated by randomly combining sequences that were selected from coding and non-coding sequences derived from the GenBank sequence database (see materials and methods) [33]. The coding and non-coding sequences had identical lengths and corresponded to a particular component of the bipartite begomovirus genome. To further evaluate the motifs that were detected in the begomovirus genomes, we introduced another value, the *mean pairwise distance D_h_* (see Materials and Methods), which described the compactness (the average similarity between the motifs in a set) of a set of motifs more precisely.

We applied all sequence sets to identify the *information content* (IC) (a ranking number assigned to each set of motifs) (Figure 3A) and to calculate the *D_h_* (Figure 3B) of every motif pair detected in the mimic genome. The results showed that the IC value of motifs detected in identical sequences was 40, whereas fewer than 0.5% of motifs detected in begomovirus-genome random mimic sequence sets and begomovirus-genome coding region mimic sequence sets had IC values larger than 37.6 and *D_h_* values less than or equal to 3 (motif length = 20 bp). To further ascertain the motif detection accuracy by MEME, we constructed a set of simulation data to help determine the threshold values of *D_h_* (Figure 3C). In the simulation, random sequences were generated and embedded with motif sets with *D_h_* values less than or equal to 3, and the simulated sequences were analyzed by MEME. We then calculated the coverage rate *C_r_* (see Materials and Methods) to estimate the fraction of embedded motif sets that were detected accurately by MEME. In the simulation, the coverage rate *C_r_* was higher than 99.8%; i.e., more than 99.8% of the motifs (with *D_h_* values less than or equal to 3) that were embedded in the simulation sequences were detected by MEME (Figure 3C). By the above evaluations, we selected motifs with IC greater than 37.6 and *D_h_* less than or equal to 3 for the analysis of bipartite begomoviruses.

The high-confidence motif allocation of all bipartite begomovirus is shown in Figure S1, and the viruses with distinctive recombination events are shown in Figure 2. The conserved region between DNA-A and DNA-B was easily detected by MEME. Moreover, some possible recombination events were also found in the unaligned region between DNA-A and DNA-B in the Potato yellow mosaic Panama virus (PYMPV), *Gossypium punctatum* mild leaf curl virus (GPMLCuV), Tomato leaf curl Hsinchu virus (ToLCHsV) and Tomato yellow leaf curl Kanchanaburi virus (TYLCKaV) (Figure 2B).

### Application of motif-based analysis to BBTV

Besides bipartite begomoviruses, we selected another multipartite virus, Banana bunchy top virus (BBTV), for our analysis. BBTV is a phytopathogenic DNA virus that causes banana bunchy top disease (BBTD). The genome of BBTV comprises at least six single-stranded, circular, integral DNA genome components, including DNA-R (encoding the replication initiation protein), -U3 (potentially encoding a protein with an unknown function), -S (encoding the capsid protein), -M (encoding a movement and RNA silencing suppressor protein), -C (encoding a cell cycle link and RNA silencing suppressor protein) and -N (encoding a nuclear shuttle protein) [34], [35], [36], [37], each of which is considered to be distinct and to share only short stretches of conserved sequences, the stem-loop (SL) and major (CR-M) common regions [35], [38], [39], [40]. We selected one isolate from each of several different geographical regions for analysis. The complete genome sequences of BBTV for each component were available from five geographical isolates (the Australia, Taiwan Type I, India Bihar, China Hainan and Tonga Tongatapu isolates) (Table 1). We also selected an Egypt Kalubia isolate that had complete sequences for DNA-R, DNA-U3, DNA-S and DNA-M but only partial sequences for DNA-C and DNA-N (Table 1).

**Table 1:** Voucher information of the BBTV sequences used in this study.

Component	Accession no.^a^	Sequence Length	CG%	Region of origin	Strain
	AusNC003479	1111	42.12%	Australia	Australia
	TaiDQ826390	1104	42.75%	Taiwan	Type 1
DNA-R	IndFJ605506	1111	42.48%	India	Bihar
	EgyAF102780	1111	42.30%	Egypt	Kalubia
	ChiAY450396	1104	43.00%	China	Hainan
	TogJF957632	1109	42.30%	Tonga	Tongatapu
	AusNC003475	1060	39.34%	Australia	Australia
	TaiDQ826392	1035	39.23%	Taiwan	Type 1
DNA-U3	IndFJ605508	1061	39.87%	India	Bihar
	EgyAF102781	929	41.55%	Egypt	Kalubia
	ChiAY606084	1067	39.55%	China	Hainan
	TogJF957644	1062	39.27%	Tonga	Tongatapu
	AusNC003473	1075	42.42%	Australia	Australia
	TaiDQ826393	1058	41.68%	Taiwan	Type 1
DNA-S	IndFJ605507	1075	42.23%	India	Bihar
	EgyAF102782	973	42.75%	Egypt	Kalubia
	ChiAY494786	1059	41.45%	China	Hainan
	TogJF957656	1075	42.42%	Tonga	Tongatapu
	AusNC003474	1043	40.46%	Australia	Australia
	TaiDQ826394	1039	39.17%	Taiwan	Type 1
DNA-M	IndFJ609642	1046	40.25%	India	Bihar
	EgyAF102783	1050	40.00%	Egypt	Kalubia
	ChiAY494788	1045	38.85%	China	Hainan
	TogJF957668	1047	40.21%	Tonga	Tongatapu
	AusNC003477	1018	39.00%	Australia	Australia
	TaiDQ826395	1014	39.15%	Taiwan	Type 1
DNA-C	IndFJ609643	1018	38.70%	India	Bihar
	EgyAF102784^b^	801	42.70%	Egypt	Kalubia
	ChiAY606085	1014	38.95%	China	Hainan
	TogJF957680	1018	38.51%	Tonga	Tongatapu
	AusNC003475	1089	38.84%	Australia	Australia
	TaiDQ826396	1086	38.40%	Taiwan	Type 1
DNA-N	IndFJ609644	1090	38.90%	India	Bihar
	EgyAF148139^b^	813	41.70%	Egypt	Kalubia
	ChiAY494787	1104	38.02%	China	Hainan
	TogJF957692	1090	38.62%	Tonga	Tongatapu

a The three-letter abbreviation of country or region names are given before accession numbers from NCBI GenBank database. (Aus, Australia; Tai, Taiwan; Ind, India; Egy, Egypt; Chi, China; Tog, Tonga).

b The BBTV component DNA5 and DNA6 of Egypt Kalubia strain (AF102784 and AF148139) are not full length sequences.

BBTV forms two groups, the Asian group (the Taiwan Type I and China Hainan isolates) and the Pacific group (the Australia, India Bihar, Egypt Kalubia and Tongatapu isolates), and our phylogenetic analysis was consistent with that grouping (Figure S2) [41], [42]. Two conserved regions, the SL (60 to 71 bp) and the CR-M region (83 to 90 bp), of the BBTV genome have been identified [35], [38], [39], [40]. Previously, the evolutionary relationships were analyzed based mainly on the SL and CR-M [35], [42]. The total length of these conserved regions represented approximately 12.9%–15.5% of the genome.

**Figure 4:**
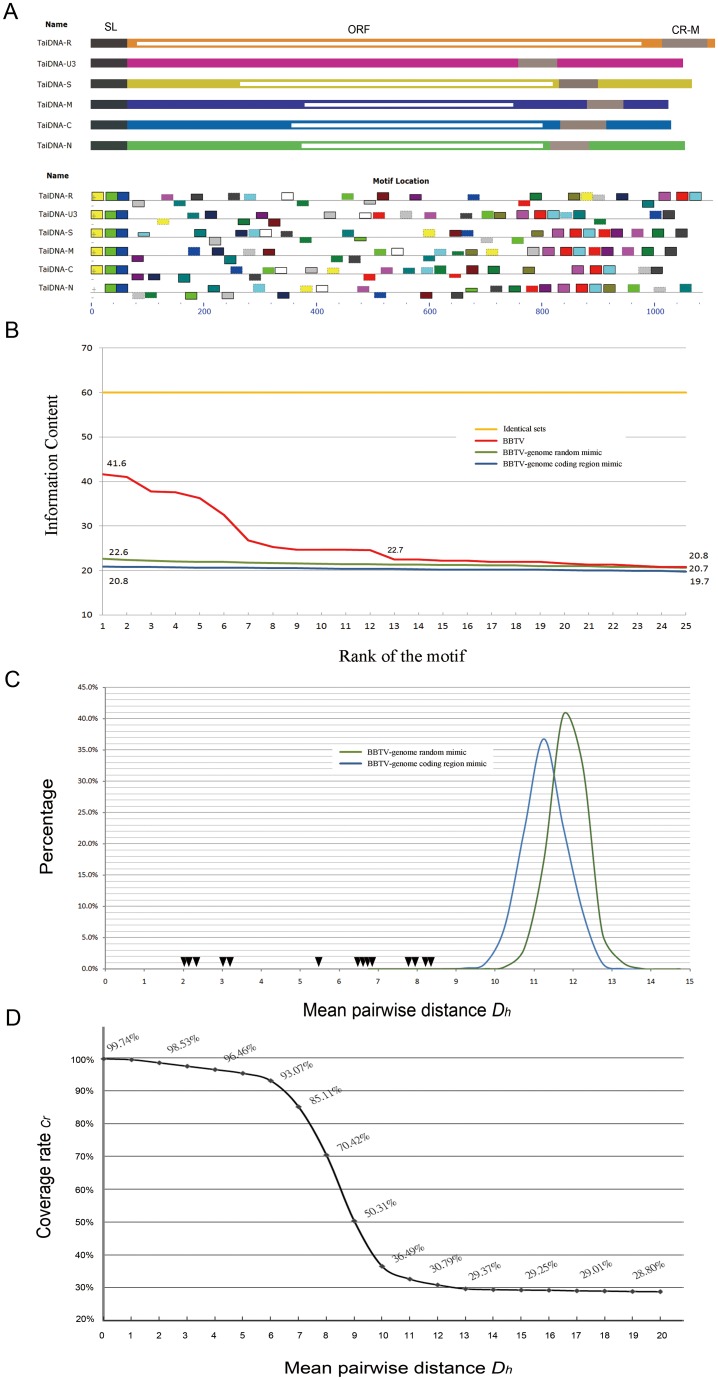
The distribution and evaluation of common motifs in the Banana bunchy top virus genome. (A) Schematic representation of the Banana bunchy top virus (BBTV) genome and the distribution of common motifs in the genomes of BBTV, detected by MEME [31]. The genome sequences of BBTV are represented by gray lines. The rectangles on the genomes represent the identified motifs. The motifs belonging to the same set are indicated in the same color. (B) The evaluation of the motifs by IC is represented on the *y*-axis. The *x*-axis represents the rank of the motif among all of the motifs identified. The red line represents the motif sets detected in the BBTV whole-genome sequences, and the green line and blue line represent the motif sets detected by the simulation of the BBTV-genome coding region mimic sequence set and the BBTV-genome random mimic sequence set, respectively. The yellow line represents the IC values that were derived from six identical sequences. (C) The distribution of the percentage of the motif sets detected from BBTV-genome mimic sequences is shown. The *x*-axis represents the mean pairwise distance (*D_h_*). The *y*-axis represents the percentage of motif sets with a certain *D_h_* value. The green line and blue line represent the distribution of the percentage of the motif sets detected by the simulation of the BBTV-genome coding region mimic sequence set and the BBTV-genome random mimic sequence set, respectively. The black rectangle represents the *D_h_* of the motif sets detected within the BBTV genome sequences. (D) The percentage of motifs detected by MEME from motifs inserted in randomly generated sequences. The *x*-axis represents the *D_h_* of artificial motifs that were generated and inserted randomly in begomovirus-genome mimic sequences The *y*-axis represents the coverage rate *C_r_* of detected motifs compared with the initially inserted artificial motifs. Only the result of the BBTV Taiwan Type I isolate is shown.

We used MEME to detect the conserved motifs between the six integral components of BBTV. The analyses were conducted for all isolates of BBTV, and for convenience, the complete analysis of the BBTV Taiwan Type I isolate is shown. The top 25 motif sets were detected (20 nucleotides in length for each motif), along with their similarity rankings (Figure 4A). Each set of motifs was assigned an IC value (Table 2). To filter out the motifs that might have significance for the evolutionary history of the virus, we also generated sequence sets as we described above, including identical sets, BBTV-genome random mimic sequence sets and BBTV-genome coding region mimic sequence sets. We compared the IC values of each motif detected from BBTV (Figure 4B, red line), identical sequence sets (Figure 4B, yellow line), BBTV-genome random mimic sequence sets (Figure 4B, blue line) and BBTV-genome coding region mimic sequence sets (Figure 4B, green line). The comparison of the IC values indicated that the motif sets with a ranking within the top 13 had a higher IC value (22.7) compared with the highest IC values derived from the BBTV-genome coding region mimic (22.6) and random mimic sequences (20.8). We also calculated the *D_h_* value of each motif set detected from the BBTV-genome coding region mimic sequence sets (Figure 4C, Blue line) and the BBTV-genome random sequence mimic sets (Figure 4C, Green line). The distributions of *D_h_* are displayed in curves with the mean values of 11.1 for the BBTV-genome coding region mimic sequence sets and 12.3 for the random mimic sequence sets. We then highlighted the *D_h_* values of 14 ranking motif sets with high IC values detected from BBTV on the curve. The results show that the *D_h_* values of the BBTV motif sets were located at the left side of the curve (Figure 4C). We also constructed a set of simulation data to ascertain the motif detection accuracy by MEME. In the simulation, random sequences were generated and embedded with motif sets with different *D_h_* values, and the simulated sequences were analyzed by MEME (Figure 4D). In the simulation, when the *D_h_* value was smaller than 8, the coverage rate *C_r_* was still higher than 70%. The C*_r_* value decreased rapidly when *D_h_* was higher than 8. When *D_h_* became greater than 9, the C*_r_* value dropped below 50.31% and then continuously approached the lower bound. Based on the simulation, we selected 8 as the threshold *D_h_* value in our study.

**Table 2:** The information content (IC) values of the top 25 motifs detected by MEME in the BBTV genomes; only the motifs detected from the Taiwan Type I isolate are shown.

Motif	*Multilevel consensus sequence	IC value
1	CGCTTAAGGGCCGCAGGCCC	41.7 bits
2	CCCCCAGCGCTCGGGACGGG	41.0 bits
3	CGGGGGTTGATTGGTCTATC	37.8 bits
4	ACGCTATGACAAAAGGGGAA	37.6 bits
5	ATGTCCCGAGTTAGTGCGCC	36.3 bits
6	AGCGCTGGGGCTTATTATTA	32.5 bits
7	CCACTTTAGTGGTGGGCCAT	26.8 bits
8	ATTCCTTGCTTCGTGACGAA	24.7 bits
9	TGAGAAGAGAAGTATATTTG	24.7 bits
10	AACAAATATACATGATACGC	24.6 bits
11	ATATATAAACAACTATGTAT	24.7 bits
12	GTGTTGAGGAAGAAAGACGC	25.3 bits
13	GCCAAGACGATGAACGGACA	22.7 bits
14	AGACGACATGAATGGATGCA	22.2 bits
15	TCGAAGGCAAAGGGAGACTT	21.9 bits
16	GCCTATAAAGAAGAGGCAGG	22.5 bits
17	TGGCAATATGTAGATTGTAT	22.2 bits
18	ATTAAAAAAGAAGAATATAA	21.3 bits
19	GAATCAAATGTAATGAATAA	20.8 bits
20	GATGAAATGATACTTTATTA	21.9 bits
21	TGACGGATAAGGATGAGACA	21.6 bits
22	ATGCTGTGACTTCCGAAGAA	21.1 bits
23	GCGCATATATTAAGAAACCA	21.3 bits
24	TTGAGTACAAGGTGAAGCC	20.8 bits
25	CCCCTCCATAACAAGATAAT	21.9 bits

* A consensus sequence of a motif set is a pseudo sequence that has a minimal average distance to every motif in the set.

### Phylogenetic analysis of BBTV

**Figure 5:**
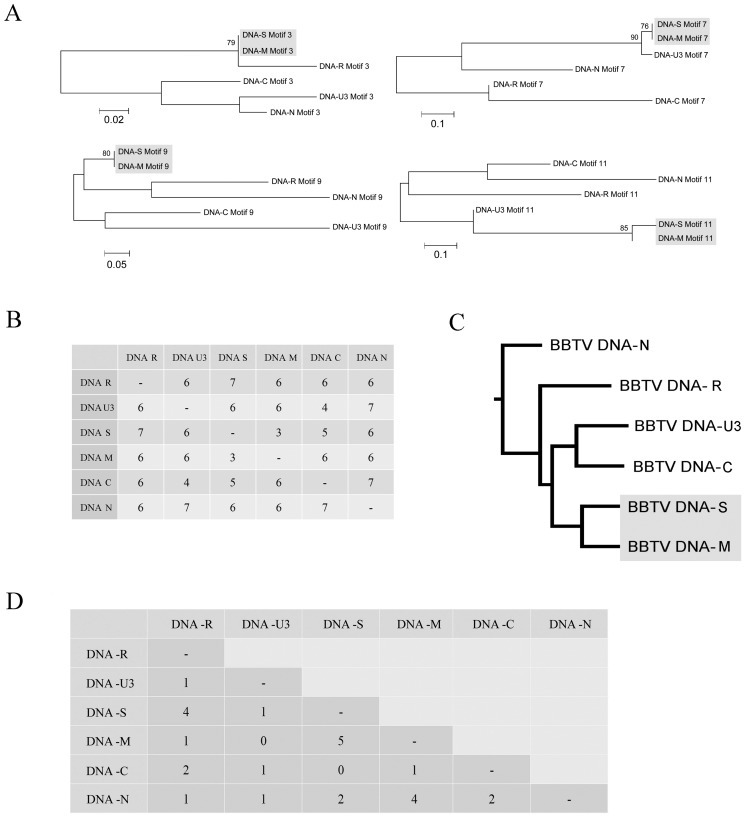
Phylogenetic analysis of a Banana bunchy top virus Taiwan isolate using motifs detected by MEME. (A) The NJ trees supported the grouping of Banana bunchy top virus (BBTV) component; only the bootstrap values above 75% were counted. (B) The distance matrix calculated by SPRING [43] represents the recombination steps that are necessary for changing the motif order from that of one genome to that of another. (C) The neighbor-joining tree constructed from the distance matrix calculated in (B). (D) The index of specifically shared motifs. The number represents the number of motifs that were shared specifically by partial components. We applied these methods to all of the BBTV isolates (Australia, India, Egypt, Taiwan, China and Tonga) (Table 3). The results derived from all of the BBTV isolates are similar, although the rearrangement distance (B) and the number of motifs that were shared specifically by subsets of components (D) varied between BBTV isolates (Figure S3). Only the result derived from the BBTV Taiwan Type I isolate is shown here.

From the above analysis, we applied the most stringent criterion (*D_h_* = 8) to select the high-confidence motifs from all of the BBTV genome components. In total, 8–12 high-confidence motifs were identified from different geographic isolates (Table 3). For every BBTV isolate, each identified high-confidence motif was studied using phylogenetic analysis. The results indicated that most of the sequences within the high-confidence motifs were similar and were phylogenetically unresolved. However, some of the motifs could be resolved (with bootstrap support >80), and the resolved phylogenies all indicated that BBTV DNA-S and DNA-M were the most closely related (Figure 5A; Table 3).

**Table 3:** The numbers of motifs detected from the genomes of five BBTV isolates and the numbers of motif trees that support the grouping of DNA-M and DNA-C with bootstrap values of above 75%.

BBTV Strain	Number of common motif sets	Number of specifically shared motif pairs	Number of trees support the grouping of DNA3 and DNA4
Taiwan: Type 1	11	23	4
Australia	11	26	5
India: Bihar	11	17	4
Egypt: Kalubia	9	24	2
China: Hainan	12	16	4
Tonga	8	12	4

In the second strategy, we applied rearrangement distance algorithms to estimate the relationships of the BBTV components based on rearrangements. The conserved motifs of each genome component were used as markers, and each sequence was represented in the form of its marker order. We used SPRING [43] to calculate the rearrangement distance between genomes. The rearrangement distance matrices of six genomes derived from different geographic isolates are shown in Figure 5B and Figure S3, and the phylogenetic tree constructed by the distance matrix is shown in Figure 5C. The motif order of DNA-S and DNA-M had the minimal rearrangement distance of all analyzed isolates (Figure 5C and S3), indicating that these two components are more closely related.

In addition to the motif sets that were shared by all of the BBTV components, we also detected conserved motifs that were shared specifically by subsets of the components of BBTV (Figure 5D and S3, Table 3). We used a relatively stringent threshold and selected only motif sets with *D_h_* values less than 6. The indices of these specifically shared motifs are shown in Figure 5D and S3. The pairs of DNA-S and DNA-M contained the largest number of specifically shared motifs (Figure 5D and S3). We conducted all of the analyses on the selected BBTV isolates; the correlation between DNA-S and DNA-M was supported by all of the methods.

### Motif distribution of BBTV

**Figure 6:**
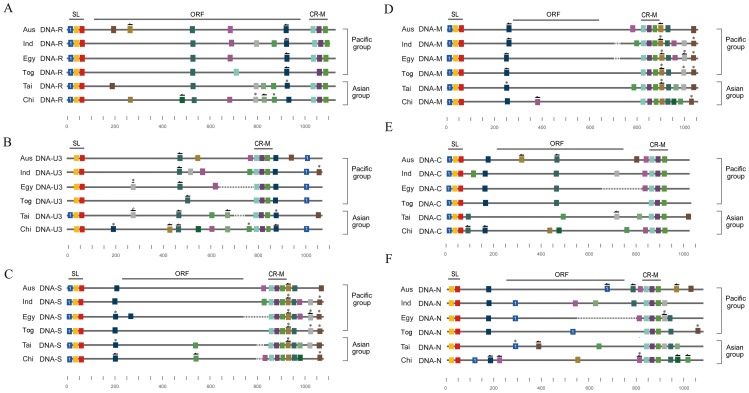
The distribution of the motifs in the genomes of five Banana bunchy top virus isolates. The solid lines represent the sequences of each Banana bunchy top virus (BBTV) genome component derived from the different isolates (Aus, Australia; Ind, India; Egy, Egypt; Tai, Taiwan, Chi, China; Tog, Tonga). All of the BBTV genome components, DNA-R (A), -U3 (B), -S (C), -M (D), -C (E), and -N (F), which were derived from different isolates, are aligned separately. The dotted lines represented gapped regions (only the gapped lengths longer than 10 are shown). The two conserved regions, the stem-loop (SL) and the major (CR-M) common region, are marked above the alignment. The colored rectangles represent the high-confidence motifs (see text) that are shared by all of the genome components of the isolates, and the motifs with similar sequences are indicated in the same color; an arrow on a rectangle indicates that the motif is reversed; a star on a rectangle indicates a high-confidence motif that is shared only by a subset of the genome components of an isolate.

We also plotted the identified high-confidence motifs on the aligned BBTV genome (Figure 6). Our results reveal several interesting phenomena. First, although the alignment of the SL region of all of the BBTV genome components showed that the SL regions of DNA-U3 and DNA-N were more distinctive than the other components (Figure S4), the SLs of DNA-U3 and -N both lacked a motif (Figure 6B and 6F). The missing motif in DNA-U3 (Figure 6B, Motif 1) was found upstream of the SL region in DNA-U3 (the BBTV genome is circular), and it was derived from all of the analyzed isolates except the Taiwan Type I isolate (Figure 6B, Motif 1). Additionally, near the CR-M regions of DNA-S and -M, there were actually long stretches of common sequences (Figure 6C and 6D). More interestingly, the detected high-confidence motifs identified surrounding the CR-M region of DNA-S and -M were also observed in the other genome components but were scattered in different positions (Figure 6).

### Application of motif-based analysis to FBNYV

**Figure 7:**
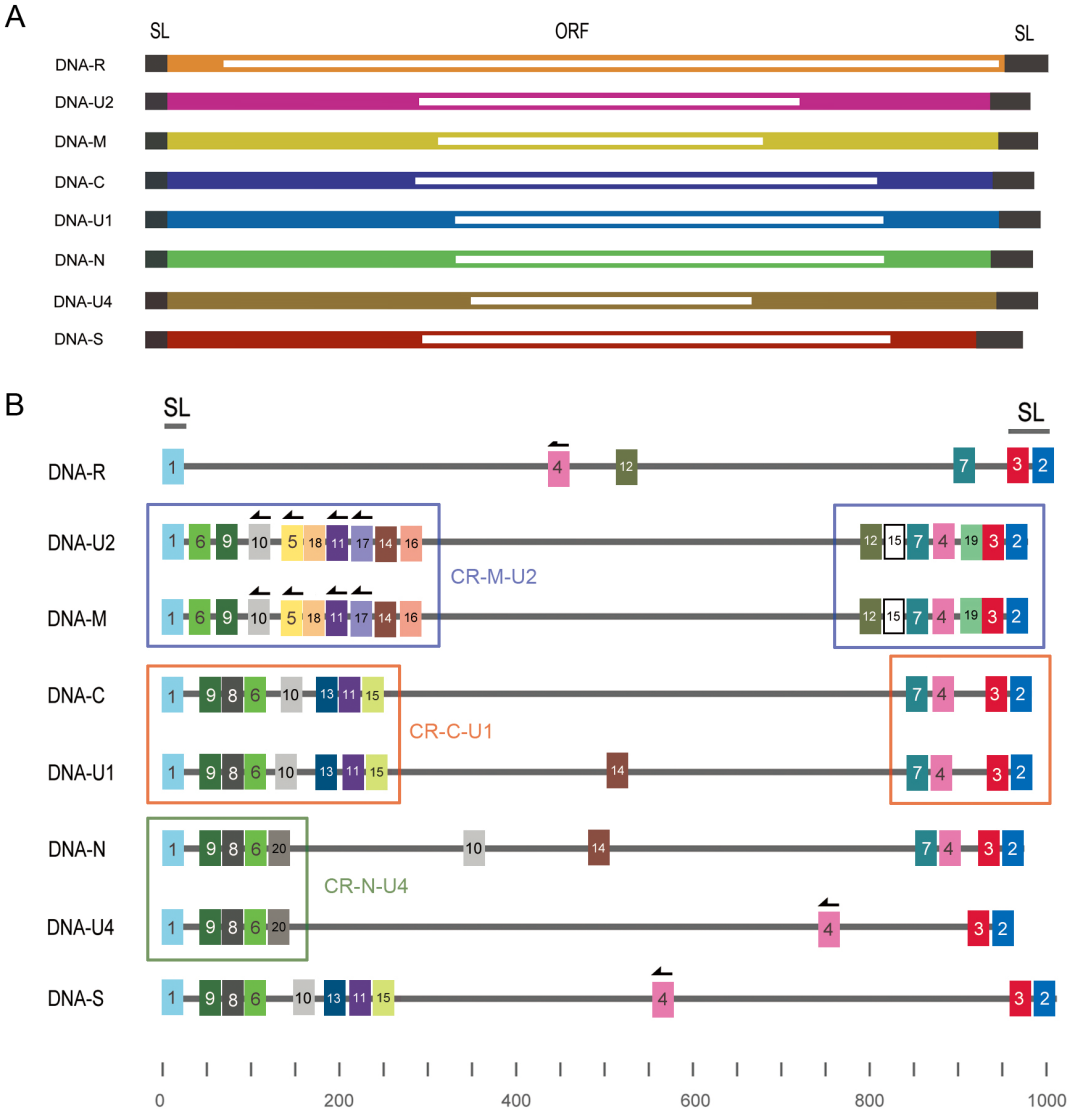
The distribution of the motifs in the genomes of Faba bean necrotic yellows virus (FBNYV). (A) Schematic representation of the Faba bean necrotic yellows virus (FBNYV) genome. (B) The distribution of common motifs in the genomes of BBTV detected by MEME. The solid lines represent the sequences of each FBNYV genome component: DNA-R, -U1, -U2, -U4, -S, -M, -C and -N. The stem-loop region of all components is marked at the top of figure. Three conserved regions, the CR-M-U2, CR-C-U1 and CR-N-U4 regions, shared only by certain components of FBNYV are also indicated. The colored rectangles represent the high-confidence motifs (see text) that are shared by genome components, and the motifs with similar sequences are indicated in the same color; an arrow on a rectangle indicates that the motif is reversed.

To demonstrate that our analytical methods can also be applied to other viruses, we selected the type species of the genus Nanovirus, Faba bean necrotic yellows virus (GQ274023–GQ274030), for analysis (Figure 7A). We used all the motif-finding and statistical analyses to identify high-confidence motifs for FBNYV (Figure S5). The motif distribution of FBNYV indicated that MEME could easily detect the known conserved region between genome components; however, our analysis revealed that several recombination events happened in these conserved regions (Figure 7B). Our analysis also revealed that several motifs, 4, 10 and 12 found in the conserved region of most genome components were rearranged in the distal regions of other genome components (Figure 7B).

## Discussion

In this study, we have developed a systematic approach to analyze the common motifs that are shared by multipartite viruses. Our methods employ motif-finding tools to detect conserved motifs from divergent genomic regions and apply statistical approaches to select high-confidence motifs. Our methods also allow us to retrieve additional information that helps to understand the evolution of viruses. We have verified the effectiveness of our approach in bipartite begomoviruses, BBTV and FBNYV, which have multi-component genomes.

Our analysis revealed that within bipartite begomoviruses, there are few common motifs can be found outside the conserved region; however, putative recombination events were still observed outside the conserved region. For example, rearrangement and duplication were found outside the previously known common region of GPMLCuV (Figure 2B). Additionally, several reversions were found between genome components of PYMPV, ToLCHsV and TYLCKaV (Figure 2B).

Our statistical analysis indicated that several short DNA motifs were scattered throughout the BBTV genome and that these motifs were not likely to have been produced by random mutation. From the distribution of detected motifs (Figure 6), we found that the distinctive SL region of DNA-U3 most likely resulted from rearranging a common motif to a different region (Figure 6 B motif 1). This suggested that the recombination events occurred in DNA-U3. Phylogenetic analysis using each of the detected common motifs derived from different DNA components also indicated that DNA-S and DNA-M are the most closely related (Figure 5A). In addition to the sequence similarity, the order of these common motifs is also the most closely related between DNA-S and DNA-M (Figure 5B and S3). Furthermore, some of the motifs that we detected are actually not shared by all of the genome components; however, DNA-S and DNA-M contain more specifically shared motifs than other DNA components (Figure 5D, Figure S3 and Table 3). Thus, the correlation between DNA-S and -M was highly supported by phylogenetic-based methods (the analysis of each motif set), rearrangement-based methods, distance-based methods and the number of specifically shared motifs (Figure 5D and Table 3). No explanation for this observation has previously reported. The distribution and arrangement of the motifs in the BBTV genome are similar in both the Pacific and the Asian groups, which suggest that the recombination event happened before the geographic separation.

Furthermore, the largest number of motifs detected in our analysis is centered near the CR-M region in DNA-S and DNA-M (Figure 6C and 6D). Endogenous primers within the BBTV virions can bind to the CR-M region and initiate the synthesis of complementary-strand DNA in vitro [37]. Interestingly, the detected motifs centered in the CR-M of DNA-S and DNA-M are also scattered within all of the other BBTV genome components (Figure 6A, 6B, 6E and 6F); whether these detected motifs are important for BBTV replication remains to be determined.

**Figure 8:**
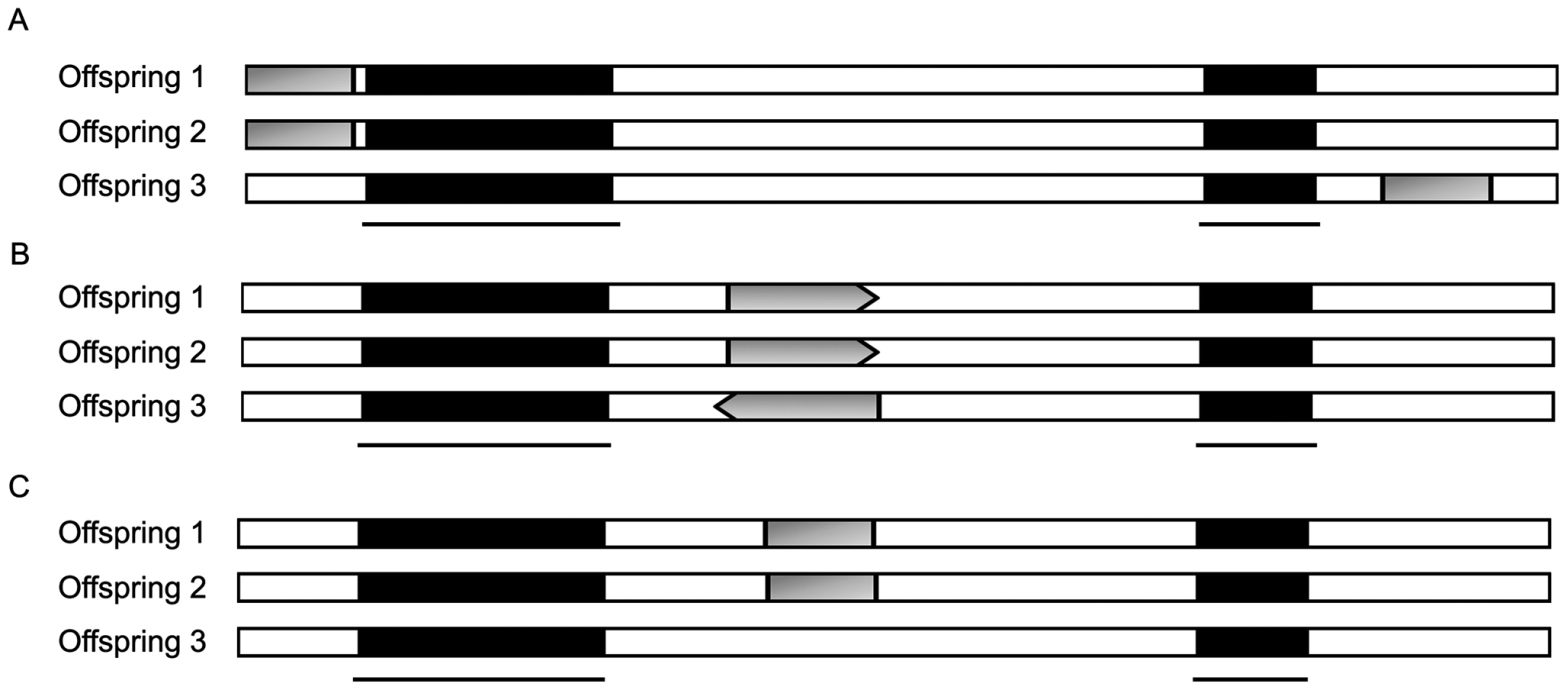
Schematic representation of the possible outcomes of genomic recombination in organisms with frequent recombination. (A) Conserved sequences (represented by dark rectangles) in the offspring genomes are separated by foreign segments (represented by empty blocks) as a result of multiple insertion events. (B) Recombination and inversion might occur in the offspring genomes and lead to positional and directional rearrangement of the conserved region (represented by dark arrows; the arrowhead indicates the direction). (C) Progeny genomes can share similar genome organization, but certain distinctive segments (represented by gray rectangles) within these regions can be shared only by a subset of the progeny.

We conclude our analysis Figure 8A–8C. In the first situation (Figure 8A), translocation of short conserved sequences occurred in all of the BBTV genome components. This result is strongly supported in BBTV DNA-U3 (Figure 6B). Reversion of conserved sequences was also identified and found to occur in all of the isolates (Figure 6). Motifs that are shared between 2–5 genome components were also frequently found (Table 3).

Previously, BBTV recombination and reassortment events have been detailed by Stainton et al. (2012) using alignment based-methods [5], and several interesting recombination events have been deduced. For example, both inter- and intra-component recombination have been found in the SL common region of DNA-U3 in some BBTV Taiwan isolates (DQ826392, DQFJ778283 and FJ463043), an India isolate (FJ009239) and a Pakistan isolate (FJ859749) [5].

Our analysis revealed that unlike other DNA-U3, DNA-U3 of the Taiwan isolate consists of 3 motifs similar to other BBTV DNA genome components. DNA-U3 found in NC003475, FJ605508, AF102781, AY606084 and JF957644 isolates only contains 2 motifs (Figure 6B). Initially, it seemed that inter-recombination happened in the SL region of DNA-U3 of the Taiwan isolate. However, alignment using DNA-U3 of DQ826392, FJ463043, FJ009239 and FJ773283 showed that SL is more conserved between isolates (Figure S6) and is less conserved between its associated DNA-R, -S, -M, -C and –N. This result suggests that intra-recombination happened in the SL region between DNA-U3 of DQ826392, FJ463043, FJ009239 and FJ773283, as previously revealed by Stainton et al. (2012).

The alignment-based methods easily identify recombination events in the conserved region, as reported by Stainton et al. (2012); however, sequences outside the conserved region, for example, the missing motif 1 in SL of DNA-U3 (Figure 6), which has relocated to a different position, will not be easily detected by alignment-based methods. Therefore, both methods are needed for a better understanding of virus evolution.

In FBNYV, we also detected some interesting rearrangement events. For example, motif 4 is located in a similar position in DNA-U1, -M, -C, -N and -U2, but reversion of motif 4 can be found in other positions in DNA-R, -S and -U4 (Figure 7B). Additionally, the distinctive conserved region consisting of motifs 7, 4, 19, 3 and 2 of DNA-U2 and DNA-M are the relocations of motif 4 within motif 3 and 7, and distinctive motifs 12, 15 and 19 are only shared by DNA-U2 and DNA-M.

Collectively, our analysis allows us to detect motifs between genome components of multipartite viruses. The analysis of these motifs allows us to reveal unusual evolution events that occurred between genome components in some begomoviruses, BBTV and FBNYV, and our data strongly suggest that genome recombination events have contributed to the evolution of BBTV integral genome components. However, we cannot rule out the possibility that some of the motifs emerged from convergent evolution. Although we interpreted these motifs from the standpoint of evolution, these motifs, which were detected as common or as partly shared motifs, could preserve function, which would allow us to further analyze their biological significance.

## Materials and Methods

### Sequences used in this study

The sequences of SPLCV, TYLCCNV and the genomes of bipartite begomoviruses used in the analysis are listed in Table S1. Six different isolates of BBTV from different geographical regions (Australia, Taiwan, India, Egypt, China and Tonga) were used (Table 1). The genome sequences of Faba bean necrotic yellows virus (GQ274023–GQ274030) were selected. The above sequences were all obtained from GenBank [33].

### Alignment, rearrangement, distance calculation, phylogenetic analysis and recombination seeking

The sequences were aligned by ClustalX 2.0 [44] using the default settings, and the rearrangement degree was estimated by SPRING (http://algorithm.cs.nthu.edu.tw/tools/SPRING/) [43], which estimates the rearrangement distance between genomes by calculating the necessary editing steps of reversals and/or block-interchanges. All of the phylogenetic trees in this study were generated by MEGA 5 [45], [46] using maximum parsimony (MP). We performed heuristic searches with 1,000 random additional replicates and tree bisection-recombination branch-swapping in the maximum parsimony analysis, and 10 trees were selected from each replicate. The branch support was estimated by PAUP version 4.0b10 [47] by bootstrapping with 1,000 replicates for both maximum parsimony and neighbor-joining (NJ) analyses. Evidence of recombination was sought by the program RDP4 (Version 4.16) [29], which implements the methods of BOOTSCAN [22], CHIMAERA [23], GENECONV [24], MAXCHI [25], RDP [26], SISCAN [27] and 3SEQ [28].

### The construction of the virus-genome mimic sequence set

The original genome sequences that were used in the sequence construction were retrieved from GenBank. The genome sequences were first processed by CDS Parser (the code can be download from http://Algorithm.cs.nthu.edu.tw/CDSParser.php) to exclude sequences that had undetermined (N) sites and to store coding regions and non-coding regions sequences separately in the database (Figure S7). We constructed virus-genome mimic sequences for the simulation of bipartite begomoviruses, BBTV and FBNYV separately. For each kind of virus, two types of virus-genome mimic sequence sets were constructed. First, a virus-genome coding region mimic sequence set was generated by simulating the virus genome, which comprises certain sequences corresponding to the mimic virus, i.e., two sequences for begomoviruses, six sequences for BBTV and eight sequences for FBNYV. Each virus-genome coding region mimic sequence was generated by combining sequences that were selected from coding and non-coding GenBank sequences, taking them from the database randomly. Each had identical lengths of coding and non-coding sequences corresponding to a particular component of the virus genome. Second, a virus-genome random mimic sequence set was also generated; each random sequence had the same length and GC content as a specific component of the virus genome.

### Motif detection and measurement of similarity

The EM-based algorithm Multiple EM for Motif Elicitation (MEME), which was introduced by Bailey and Elkan [30], [48], was used for the detection of sequence motifs in the virus genomes (begomoviruses, BBTV and FBNYV) used in this study. The genome sequence of each virus was submitted to MEME (http://meme.nbcr.net/meme/) [31] for the determination of similar segments (motifs). The variables used in MEME are listed in Table 4. The *information content value* (IC, the relative entropy of the motif relative to a uniform background frequency model) of each motif set was calculated. Pairwise motif correlations were checked by MAST [49], [50] to exclude similar motif pairs (correlation >0.60) and to identify the corresponding positions of each motif in the virus sequences.

**Table 4:** The variables used in MEME to detect motifs.

Variables	Command	Value
Sequence use DNA alphabet	-DNA	–
Distribution of motifs	-mod	oops
Maximum number of motifs to find	-nmotifs	25
Stop if motif E-value greater than <evt>	-evt	1e+100
Minimum motif width	-minw	20
Maximum motif width	-maxw	20
Stop if motif IC lower than	-minic	0.0
Maximum number of sites for each motif	-maxsites	10
Weight on expected number of sites	-wnsites	0.8

The measure of similarity for each motif set was defined as the *mean pairwise distance* (MPD) or *D_h_* to further verify the confidence of each motif set detected by MEME. For every set of motifs, we calculated the Hamming distance [51] *h* between each pair of motifs in the set as the first step. Then, the distance *h* between each motif pair was summed and divided by the total number of pairs (*n* motifs give 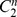 = *n(n-1)/2* possible pairs) to calculate the *D_h_* score. The score *D_h_* of the motif set *M* can be written as:
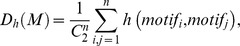
where *n* is the number of motifs in motif set *m*. The *D_h_* score for each motif set act as a normalized value for variation, in which low *D_h_* scores correspond to high conservation between motifs. The observed *D_h_* values theoretically reflect the compactness of the motif set *m*.

### Simulation data construction for threshold determination

The procedure for constructing the simulation data includes 2 steps. In the first step, 1,000 sequence sets *G_1_–G_1000_* are constructed, and each set *G_i_* contains *n* sequences *S_1_–S_n_*, where *n* = 2, 6 and 8 for the simulation of the bipartite begomovirus, BBTV and FBNYV genomes, respectively. Each sequence in the set (*S_j_*) was constructed randomly. However, the length and GC content of each sequence in each set (*G_i_*) were equal to those in each corresponding virus genome component.

In the second step, for each sequence set *G_i_* in step 1, we randomly constructed 20 sets of motifs *M_1_–M_20_*, each of which contained *n* motifs *m_1_–m_n_* that were designed to have the *D_h_* value *d*. Then, each *m_i_* of *M_1_–M_20_* replaced a random subsequence that was located in *S_i_* and had the same length as *m_i_*. We also reversed the inserted motif randomly to reflect the sequence reversal events caused by recombination.

The sequence construction in the second step was repeated 10,000 times with a *D_h_* value *d* ranging from 0 to 4, 20 for the simulation of the bipartite begomovirus, BBTV and FBNYV genomes, respectively. We also constructed 10,000 supplementary sequence sets that were embedded with random motif sets (*d* = ∞) for contrast. Each simulation sequence of set *G* was submitted to MEME for motif detection with the variables listed in Table 4. To estimate the percentage of embedded motif sets (*M_c_*) that could be detected accurately by MEME under different *d* values, we defined a normalized measure of the coverage ratio *C_r_* as
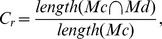
where *M_d_* is the set of motif sets detected by MEME.

## Supporting Information

Figure S1 **The high-confidence motifs detected between genomes of bipartite begomovirus.** The gray line represents the genome sequence, and common motifs detected by MEME are indicated by colored squares. Motifs located below the gray line indicate that the motifs are reversed, and the motifs belonging to the same set in the same genome are indicated in the same color.
(TIF)

Figure S2 **Phylogram of the genome sequences of Banana bunchy top virus.** (A–F) Phylograms of the maximum-parsimony trees based on the total nucleotide sequences (DNA-R, -U3, -S, -M, -C and -N) from Banana bunchy top virus (BBTV) geographic isolates (Australia, Taiwan, India, China, Egypt and Tonga, respectively). In the phylograms in E and F, the component sequences of the Egypt Kalubia strain (AF102784 and AF148139) were excluded from the phylogenetic analysis because they were not full-length sequences (see Table 1). Along the branches are the bootstrap supports of the maximum-parsimony and neighbor-joining methods; only values >70% are shown. For the BBTV integral components, the sequences were derived from isolates for which all of the integral-component sequences were available in GenBank.
(TIF)

Figure S3 **Phylogenetic analysis of the genome of Banana bunchy top virus using motifs detected by MEME.** (A) The index of specifically shared motifs derived from the Banana bunchy top virus (BBTV) isolates (Australia, India, Egypt, China and Tonga). The number represents the number of motifs that were shared between paired genome components. (B) The distance matrix calculated by SPRING [43] represents the number of recombination steps necessary to change the motif order from that of one component genome to that of another.
(TIF)

Figure S4 **Alignment of the stem-loop common region of Banana bunchy top virus.** The sequence alignments of the stem-loop region derived from the Banana bunchy top virus (BBTV) genome components of the Pacific group (Australia, India Bihar, Egypt Kalubia) and the Asian group of isolates (Taiwan Type I, China Hainan). Identical and conserved sequences within the alignment are indicated in black and gray shadow, respectively.
(TIF)

Figure S5 **The simulation result of Faba bean necrotic yellows virus (FBNYV).** (A) The evaluation of the motifs detected in FBNYV using the information content (IC) is represented. (B) The distribution of the percentage of the motif sets detected from FBNYV-genome mimic sequences. (C) The percentage of motifs detected by MEME [31] from motifs inserted in randomly generated sequences. The strategies are similar to Figure 4B, 4C and 4D in the analysis of BBTV.
(TIF)

Figure S6 **Alignment of the stem-loop common region of Banana bunchy top virus.** The sequence alignments of the stem-loop region derived from the Banana bunchy top virus (BBTV) genome components of Taiwan, India and Pakistan isolates.
(TIF)

Figure S7 **Schematic diagram of the construction of a BBTV-genome coding region mimic sequence set.**
(TIF)

Table S1 **List of begomoviruses used in this study.**
(DOCX)
