# Supplementary figures and images for: Application of Motif-Based Tools on Evolutionary Analysis of Multipartite Single-Stranded DNA Viruses
Source: PLoS One. 2013 Aug 6;8(8):e71565. doi: 10.1371/journal.pone.0071565 (PMC3735576; doi:10.1371/journal.pone.0071565)

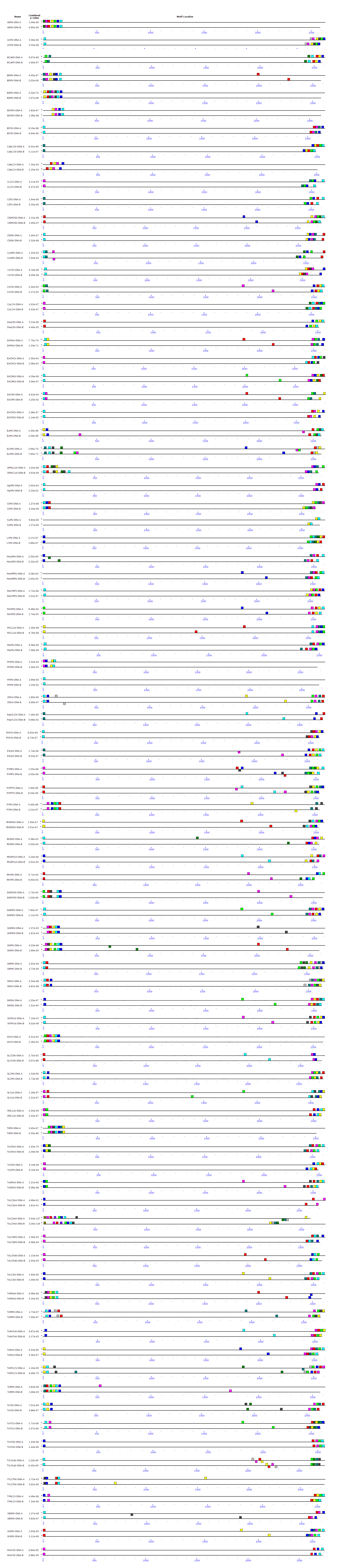

Supplement: Figure S1 — The high-confidence motifs detected between genomes of bipartite begomovirus. The gray line represents the genome sequence, and common motifs detected by MEME are indicated by colored squares. Motifs located below the gray line indicate that the motifs are reversed, and the motifs belonging to the same set in the same genome are indicated in the same color. (TIF) [file pone.0071565.s001.tif]

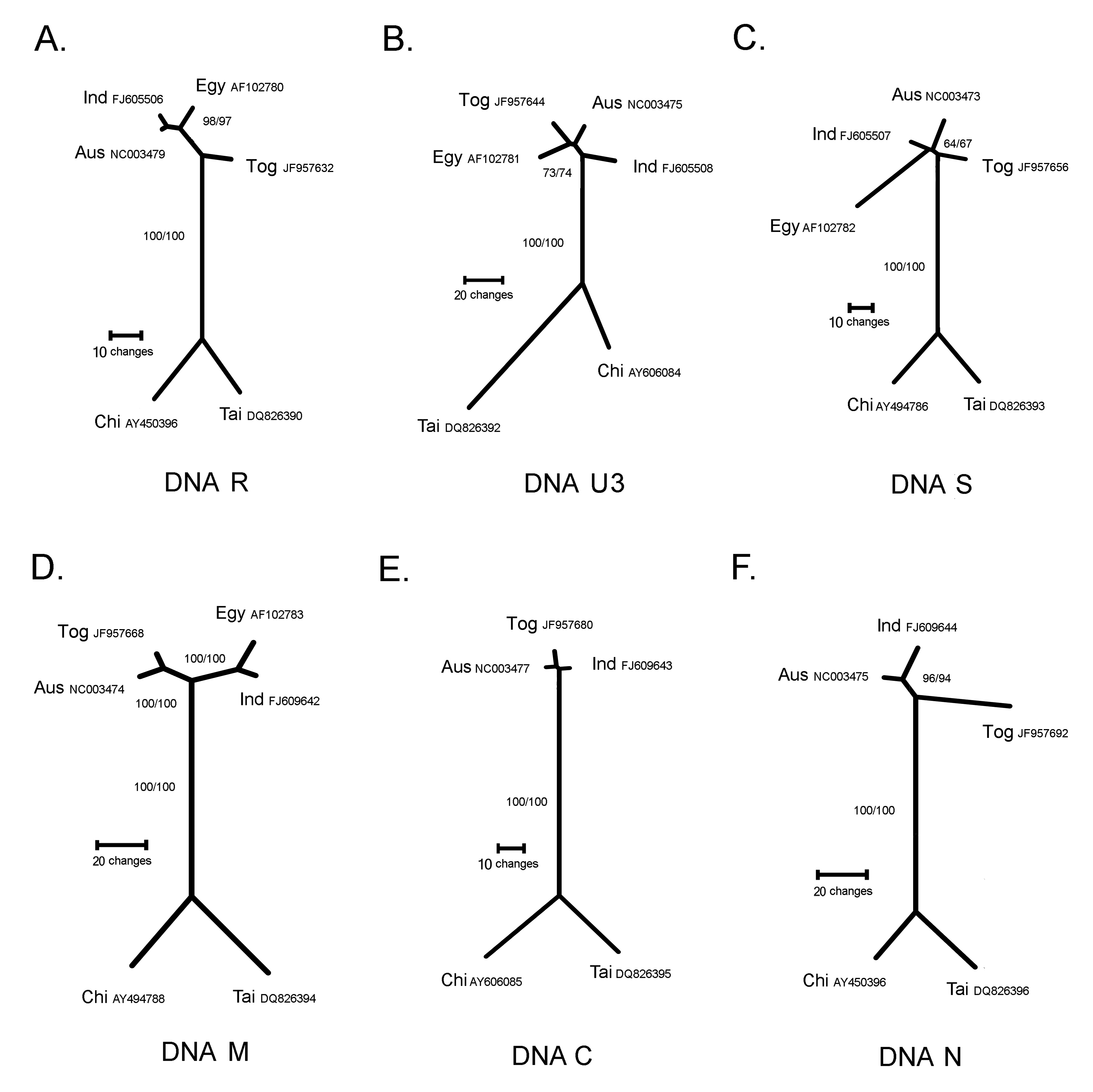

Supplement: Figure S2 — Phylogram of the genome sequences of Banana bunchy top virus. (A–F) Phylograms of the maximum-parsimony trees based on the total nucleotide sequences (DNA-R, -U3, -S, -M, -C and -N) from Banana bunchy top virus (BBTV) geographic isolates (Australia, Taiwan, India, China, Egypt and Tonga, respectively). In the phylograms in E and F, the component sequences of the Egypt Kalubia strain (AF102784 and AF148139) were excluded from the phylogenetic analysis because they were not full-length sequences (see Table 1). Along the branches are the bootstrap supports of the maximum-parsimony and neighbor-joining methods; only values >70% are shown. For the BBTV integral components, the sequences were derived from isolates for which all of the integral-component sequences were available in GenBank. (TIF) [file pone.0071565.s002.tif]

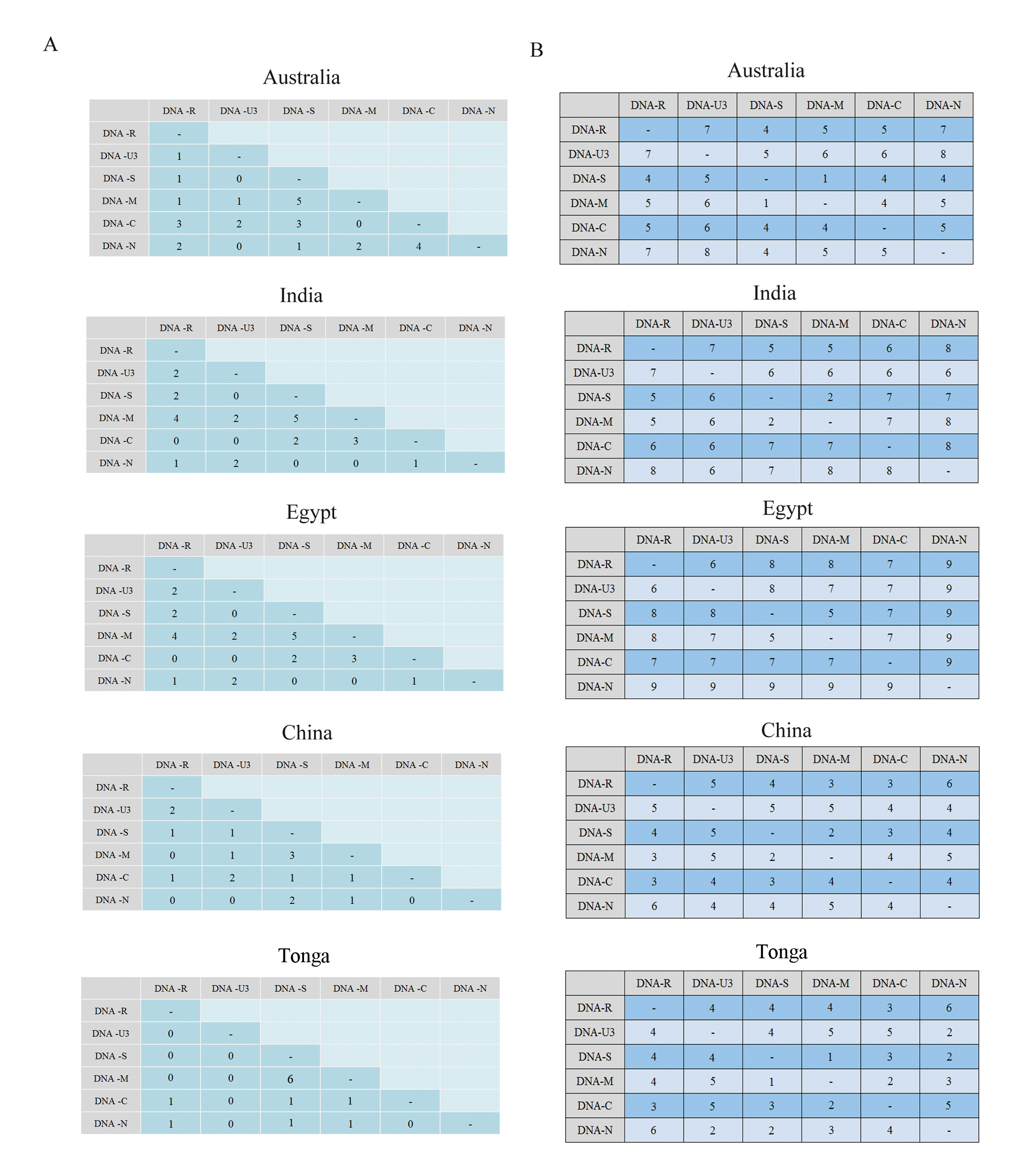

Supplement: Figure S3 — Phylogenetic analysis of the genome of Banana bunchy top virus using motifs detected by MEME. (A) The index of specifically shared motifs derived from the Banana bunchy top virus (BBTV) isolates (Australia, India, Egypt, China and Tonga). The number represents the number of motifs that were shared between paired genome components. (B) The distance matrix calculated by SPRING [43] represents the number of recombination steps necessary to change the motif order from that of one component genome to that of another. (TIF) [file pone.0071565.s003.tif]

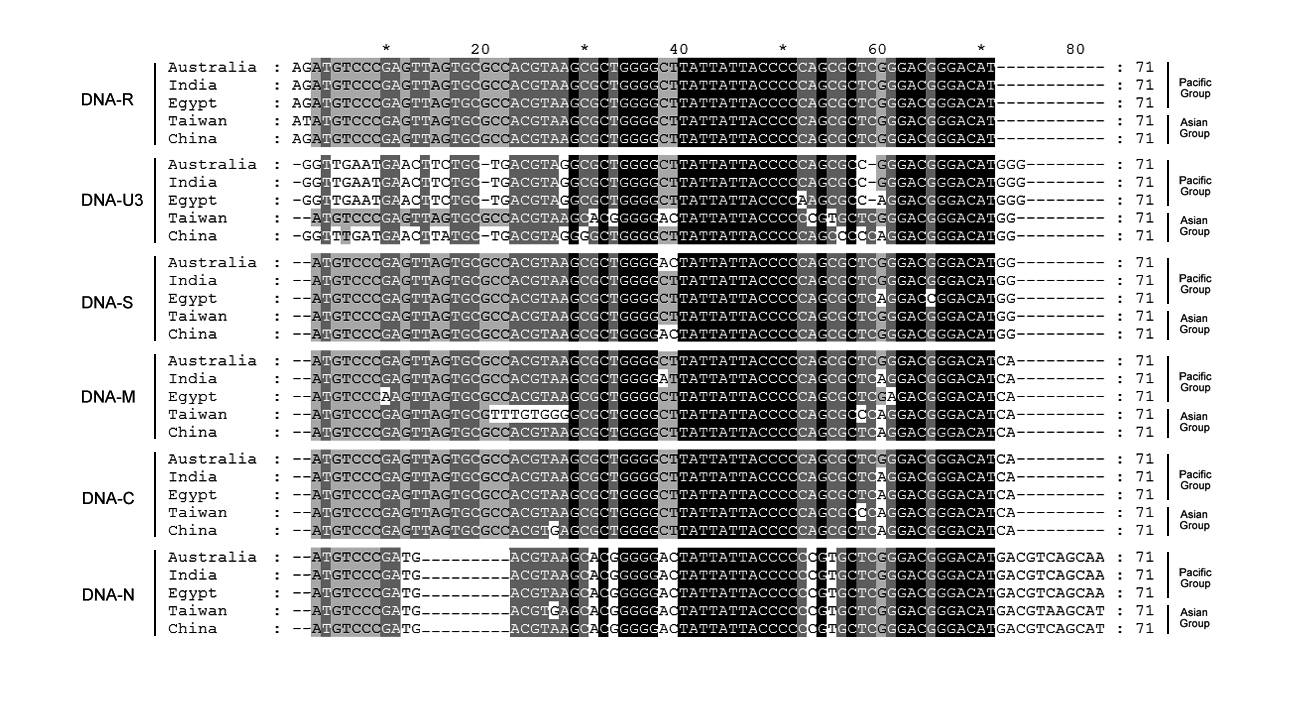

Supplement: Figure S4 — Alignment of the stem-loop common region of Banana bunchy top virus. The sequence alignments of the stem-loop region derived from the Banana bunchy top virus (BBTV) genome components of the Pacific group (Australia, India Bihar, Egypt Kalubia) and the Asian group of isolates (Taiwan Type I, China Hainan). Identical and conserved sequences within the alignment are indicated in black and gray shadow, respectively. (TIF) [file pone.0071565.s004.tif]

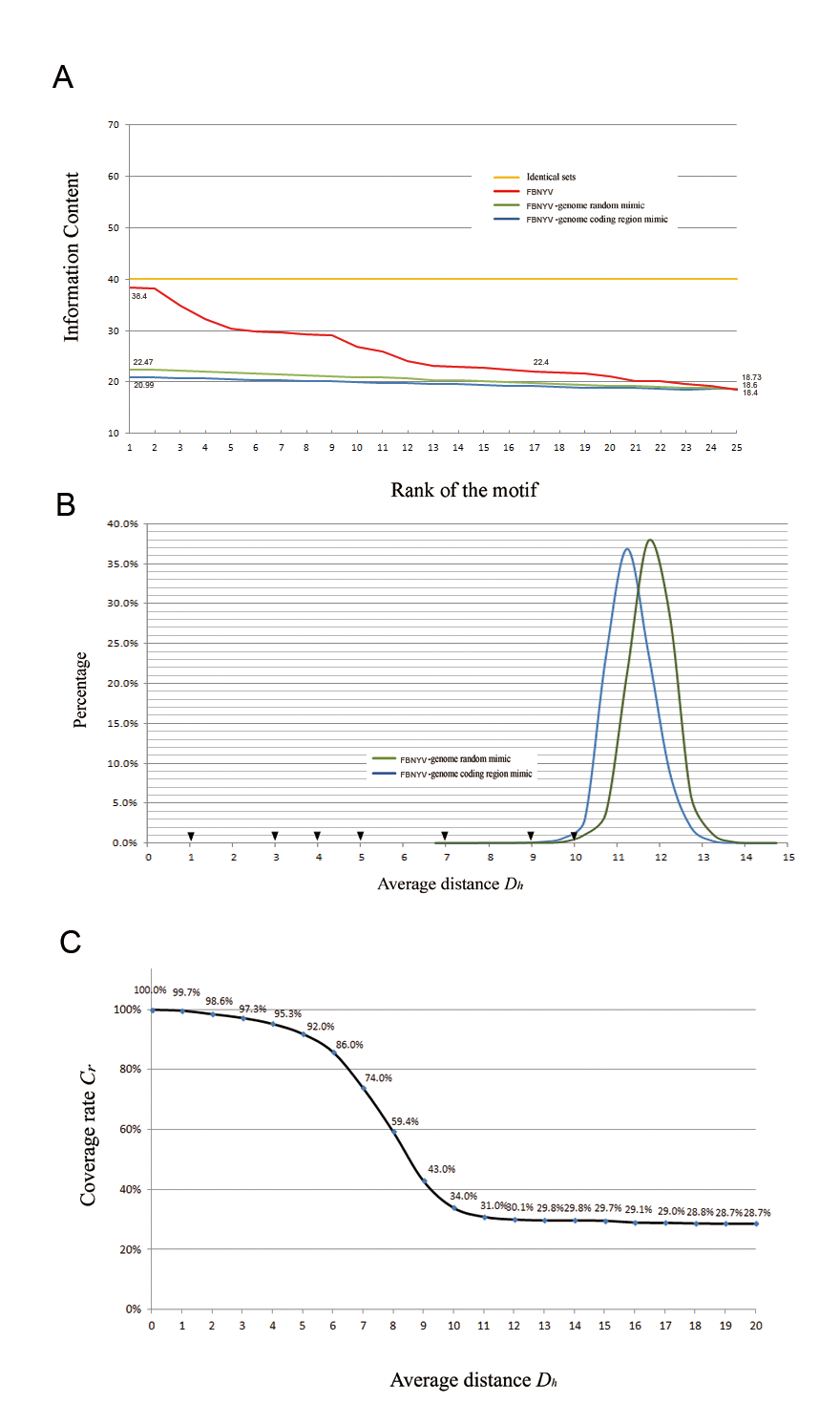

Supplement: Figure S5 — The simulation result of Faba bean necrotic yellows virus (FBNYV). (A) The evaluation of the motifs detected in FBNYV using the information content (IC) is represented. (B) The distribution of the percentage of the motif sets detected from FBNYV-genome mimic sequences. (C) The percentage of motifs detected by MEME [31] from motifs inserted in randomly generated sequences. The strategies are similar to Figure 4B, 4C and 4D in the analysis of BBTV. (TIF) [file pone.0071565.s005.tif]

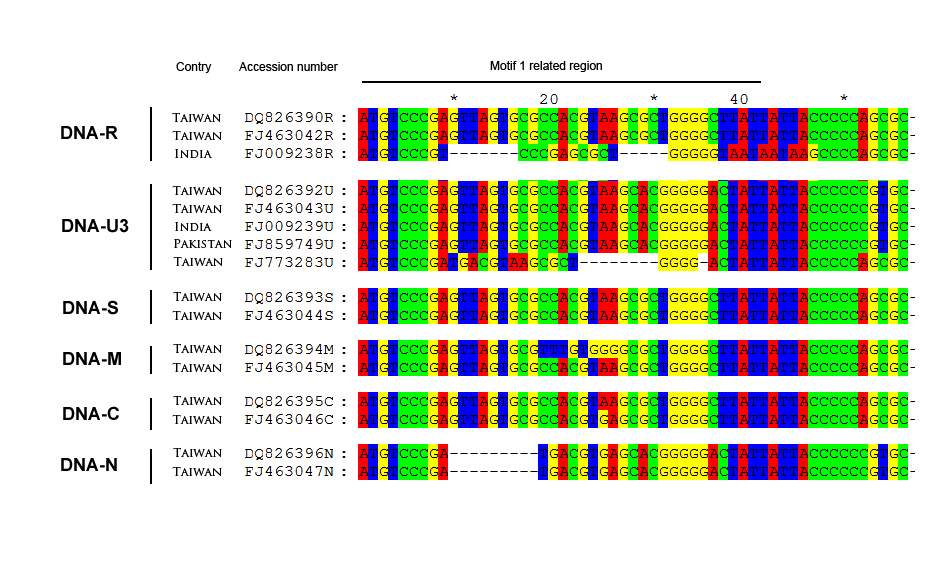

Supplement: Figure S6 — Alignment of the stem-loop common region of Banana bunchy top virus. The sequence alignments of the stem-loop region derived from the Banana bunchy top virus (BBTV) genome components of Taiwan, India and Pakistan isolates. (TIF) [file pone.0071565.s006.tif]

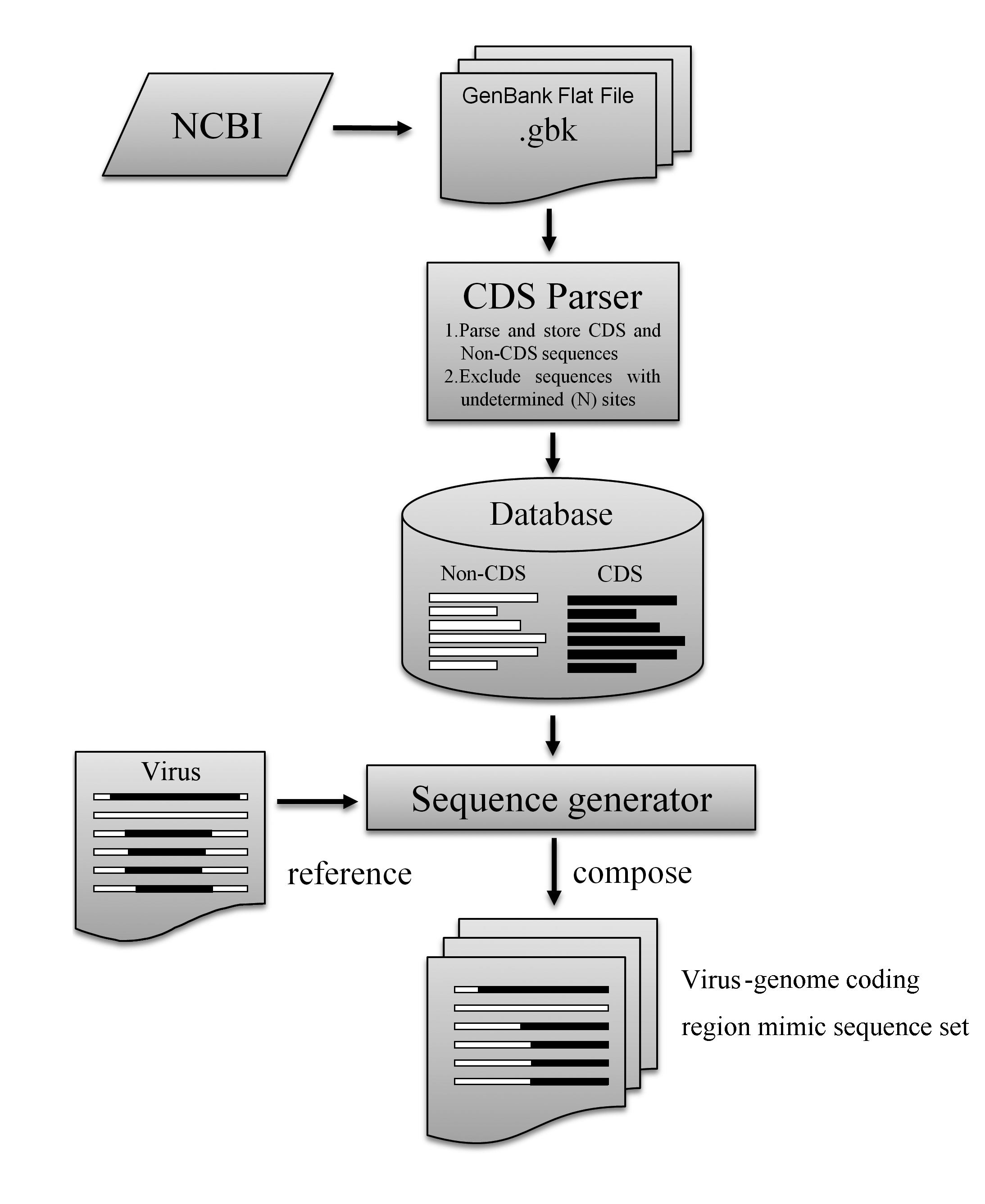

Supplement: Figure S7 — Schematic diagram of the construction of a BBTV-genome coding region mimic sequence set. (TIF) [file pone.0071565.s007.tif]
