# Supplementary material for: Application of Motif-Based Tools on Evolutionary Analysis of Multipartite Single-Stranded DNA Viruses
Source: PLoS One. 2013 Aug 6;8(8):e71565. doi: 10.1371/journal.pone.0071565 (PMC3735576; doi:10.1371/journal.pone.0071565)
Supplement: Table S1 — List of begomoviruses used in this study. (DOCX) [file pone.0071565.s008.docx]

Table S1. List of begomoviruses used in this study

A. List of monopartite geminiviruses and their associated satellite viruses used in this study

| **Name** | **Sequence** | **Accession number** |
| --- | --- | --- |
| Sweet leaf curl virus | SPLCV-BR | HQ393455 |
|  | SPLCV-SP | HQ393473 |
|  | SPLCV-US | HQ393450 |
| Tomato leaf curl China virus | DNA-A | AJ31967 |
|  | DNA-*β* | AJ421621 |
|  | RecDNA-A*β* | AJ781297 |
|  |  |  |

B. List of bipartite viruses in the genus Begomovirus used in this study

| **Name** | **Accession number** | | **abbreviation** |
| --- | --- | --- | --- |
|  | DNA-A | DNA-B |  |
| ***Abutilon mosaic virus***  Abutilon mosaic virus - Germany | X15983 | X15984 | AbMV |
| ***African cassava mosaic virus***  African cassava mosaic virus - Cameroon | AF112352 | AF112353 | ACMV |
| ***Bean calico mosaic virus***  Bean calico mosaic virus - Mexico | AF110189 | AF110190 | BCaMV |
| ***Bean dwarf mosaic virus***  Bean dwarf mosaic virus - Colombia | M88179 | M88180 | BDMV |
| ***Bean golden mosaic virus***  Bean golden mosaic virus - Brazil | M88686 | M88687 | BGMV |
| ***Bean golden yellow mosaic virus***  Bean golden yellow mosaic virus - Dominican | L01635 | L01636 | BGYMV |
| ***Cabbage leaf curl Jamaica virus***  Cabbage leaf curl Jamaica virus - Jamaica | DQ178608 | DQ178609 | CabLCJV |
| ***Cabbage leaf curl virus***  Cabbage leaf curl virus - United state | U65529 | U65530 | CabLCV |
| ***Chino del tomate virus***  Chino del tomate virus - Maxico | AF101476 | AF101478 | CdTV |
| ***Clerodendron golden mosaic virus***  Clerodendron golden mosaic virus - Vietnam | DQ641692 | DQ641693 | ClGNV |
| ***Corchorus golden mosaic virus***  Corchorus golden mosaic virus - India | FJ463902 | FJ463901 | CoGMV |
| ***Corchorus yellow spot virus***  Corchorus yellow spot virus - Mexico | DQ875868 | DQ875869 | CoYSV |
| ***Corchorus yellow vein virus***  Corchorus yellow vein virus - Vietnam | AY727903 | AY727904 | CoTVV |
| ***Cotton leaf crumple virus***  Cotton leaf crumple virus - Mexico | AF480940 | AF480941 | CLCrV |
| ***Cucurbit leaf crumple virus***  Cucurbit leaf crumple virus – United state | AF256200 | AF327559 | CuLCrV |
| ***Desmodium leaf distortion virus***  Desmodium leaf distortion virus - Mexico | DQ875870 | DQ875871 | DesLDV |
| ***Dicliptera yellow mottle virus***  Dicliptera yellow mottle virus – United state | AF139168 | AF170101 | DiYMoV |
| ***East African cassava mosaic Cameroon virus***  East African cassava mosaic Cameroon virus - Cameroon | AF112354 | AF112355 | EACMCV |
| ***East African cassava mosaic Kenya virus***  East African cassava mosaic Kenya virus - Kenya | AJ717580 | AJ704965 | EACMKV |
| ***East African cassava mosaic virus***  East African cassava mosaic virus - Kenya | AJ717542 | AJ704949 | EACMV |
| ***East African cassava mosaic Zanzibar virus***  East African cassava mosaic Zanzibar virus - Kenya | AJ717562 | AJ704942 | EACMZV |
| ***Euphorbia mosaic virus***  Euphorbia mosaic virus - Cuba | FJ807782 | FJ807783 | EuMV |
| ***Horsegram yellow mosaic virus***  Horsegram yellow mosaic virus - India | AM932427 | AM932428 | HgYMV |
| ***Indian cassava mosaic virus***  Indian cassava mosaic virus India | AY730035 | AY730036 | ICMV |
| ***Kudzu mosaic virus***  Kudzu mosaic virus - China | FJ539014 | FJ539015 | KuMV |
| ***Luffa yellow mosaic virus***  Luffa yellow mosaic virus -Vietnam | AF509739 | AF509740 | LYMV |
| ***Macroptilium mosaic Puerto Rico virus***  Macroptilium mosaic Puerto Rico virus – Purto Rico | AY044133 | AY044134 | MacMPRV |
| ***Macroptilium yellow mosaic Florida virus***  Macroptilium yellow mosaic Florida virus - United state | AY044135 | AY044136 | MacYMFV |
| ***Melon chlorotic leaf curl virus***  Melon chlorotic leaf curl virus – Costa Rica | AY064391 | AF440790 | MCLCuV |
| ***Mungbean yellow mosaic India virus***  Mungbean yellow mosaic India virus – India | AF416742 | AF416741 | MYMIV |
| ***Mungbean yellow mosaic virus***  Mungbean yellow mosaic virus - India | AJ421642 | AJ867554 | MYMV |
| ***Pepper huasteco yellow vein virus***  Pepper huasteco yellow vein virus - Mexico | GU128150 | GU128146 | PHYVV |
| ***Pepper yellow leaf curl Indonesia virus***  Pepper yellow leaf curl Indonesia virus - Indonesia | AB267834 | AB267835 | PepYLCIV |
| ***Potato yellow mosaic Panama virus***  Potato yellow mosaic Panama virus - Panama | Y15034 | Y15033 | PYMPV |
| ***Potato yellow mosaic virus***  Potato yellow mosaic virus - Venezuela | D00940 | D00941 | PYMV |
| ***Rhynchosia golden mosaic Sinaloa virus***  Rhynchosia golden mosaic Sinaloa virus - Mexico | DQ406672 | DQ406673 | RhGMSiV |
| ***Rhynchosia golden mosaic virus***  Rhynchosia golden mosaic virus -Mexico | DQ347950 | DQ356429 | RhGMV |
| ***Sida golden mosaic Costa Rica virus***  Sida golden mosaic Costa Rica virus – Costa Rica | X99550 | X99551 | SiGMCRV |
| ***Sida golden mosaic Florida virus***  Sida golden mosaic Florida virus -Cuba | HM003779 | HM003778 | SiGMFiV |
| ***Sida golden mosaic Honduras virus***  Sida golden mosaic Honduras virus - Honduras | Y11097 | Y11098 | SiGMHV |
| ***Sida golden mosaic virus***  Sida golden mosaic virus – United states | AF049336 | AF039841 | SiGMV |
| ***Sida micrantha mosaic virus***  Sida micrantha mosaic virus - Brazil | AJ557451 | AJ557453 | SiMMV |
| ***Sida mottle virus***  Sida mottle virus - Brazil | AJ557450 | AJ557454 | SiMoV |
| ***Sida yellow mosaic Yucatan virus***  Sida yellow mosaic Yucatan virus - Mexico | DQ875872 | DQ875873 | SiYMYuV |
| ***Sida yellow vein virus***  Sida yellow vein virus -Honduras | Y11099 | Y11100 | SiYVV |
| ***Squash leaf curl China virus***  Squash leaf curl China virus -China | AM260206 | AM260208 | SLCCNV |
| ***Squash leaf curl virus***  Squash leaf curl virus - Jordan | EF532620 | EF532621 | SLCuV |
| ***Squash mild leaf curl virus***  Squash mild leaf curl virus – United states | AF421552 | AF421553 | SMLCuV |
| ***Sri Lankan cassava mosaic virus***  Sri Lankan cassava mosaic virus - India | AJ579307 | AJ579308 | SLCMV |
| ***Tomato chlorotic mottle virus***  Tomato chlorotic mottle virus -Brazil | AF490004 | AF491306 | ToCMoV |
| ***Tomato golden mosaic virus***  Tomato golden mosaic virus -Brazil | K02029 | K02030 | TGMV |
| ***Tomato golden mottle virus***  Tomato golden mottle virus - Mexico | DQ520943 | DQ406674 | ToGMoV |
| ***Tomato leaf curl Gujarat virus***  Tomato leaf curl Gujarat virus - India | AY190290 | AY190291 | ToLCGuV |
| ***Tomato leaf curl Hsinchu virus***  Tomato leaf curl Hsinchu virus - China | EU596959 | EU596960 | ToLCHsV |
| ***Tomato leaf curl New Delhi virus***  Tomato leaf curl New Delhi virus - Bangladesh | AJ875157 | AJ875158 | ToLCNDV |
| ***Tomato leaf curl Sinaloa virus***  Tomato leaf curl Sinaloa virus - Nicaragua | AJ608286 | AJ508783 | ToLCSiV |
| ***Tomato mild yellow leaf curl Aragua virus***  Tomato mild yellow leaf curl Aragua virus - Venezeula | AY927277 | EF547938 | ToMYLCV |
| ***Tomato mosaic Havana virus***  Tomato mosaic Havana virus - Cuba | Y14874 | Y14875 | ToMHaV |
| ***Tomato mosaic leaf curl virus***  Tomato mosaic leaf curl virus - Puerto | AF068636 | AY965899 | MerMV |
| ***Tomato mottle Taino virus***  Tomato mottle Taino virus -Cuba | AF012300 | AF012301 | ToMoTaV |
| ***Tomato mottle virus***  Tomato mottle virus | AY965900 | AY965901 | ToMoV |
| ***Tomato rugose mosaic virus***  Tomato rugose mosaic virus - Brazil | AF291705 | AF291706 | ToRMV |
| ***Tomato yellow leaf curl Kanchanaburi virus***  Tomato yellow leaf curl Kanchanaburi virus -Thailand | AF511529 | AF511528 | TYLCKaV |
| ***Tomato yellow leaf curl Thailand virus***  Tomato yellow leaf curl Thailand virus - Thailand | X63015 | X63016 | TYLCTHV |
| ***Tomato yellow margin leaf curl virus***  Tomato yellow margin leaf curl virus Vinezuela | AY508993 | AY508994 | TYMLCV |
| ***Tomato yellow spot virus***  Tomato yellow spot virus -Brazil | DQ336350 | DQ336351 | ToYSV |
| ***Tomato yellow vein streak virus***  Tomato yellow vein streak virus -India | EF417915 | EF417916 | ToYVSV |
| ***Watermelon chlorotic stunt virus***  Watermelon chlorotic stunt virus - Iran | AJ245652 | AJ245653 | WmCSV |
|  |  |  |  |

* Species names are in bold italic scrip and names of strains are in roman script.

C. List of possible bipartite Begomoviruses that have not been approved as species..

| **Name** | **Accession number** | | **abbreviations** |
| --- | --- | --- | --- |
|  | DNA-A | DNA-B |  |
| Blainvilea yellow spot virus | EU710756 | EU710757 | BIYSV |
| Clerodendron golden mosaic virus | FJ011668 | FJ011669 | ClGMCNV |
| Euphoribia yellow mosaic virus | FJ619507 | FJ619508 | EuYMV |
| Gossypium punctatum mild leaf curl virus | EU384575 | EU384578 | GPMLCuV |
| Macroptilium golden mosaic virus | EU158096 | EU158097 | MacGMV |
| Malvastrum yellow vein Jamaica virus | FJ601917 | FJ600485 | MaYMJV |
| Okra mottle virus | EU914817 | EU914818 | OMoV |
| Passionfruit severe leaf distortion virus | FJ972767 | FJ972768 | PSLDV |
| Potatto yellow mosaic Trinidad virus | AF039031 | AF039032 | PYMTTV |
| Rhynchosia golden mosaic Yucatan virus | EU021216 | FJ792608 | RhGMYuV |
| Rhynchosia yellow mosaic virus | AM999981 | AM999982 | RhYMV |
| Sida mosaic Sinaloa virus | DQ520944 | DQ356428 | SiMSiV |
| Tomato common mosaic virus | EU710754 | EU710755 | ToCMV |
| Tomato leaf curl Palampur virus | AM884015 | AM992534 | ToLCPalV |
| Tomato mild mosaic virus | EU710752 | EU710753 | ToMMV |
| Tomato yellow distortion leaf virus | FJ174698 | FJ999999 | ToYTLV |
| Velvet bean severe mosaic virus | FN543425 | FN543426 | VBSMV |
| Wissadula golden mosaic virus | GQ355488 | GQ355487 | WGMV |
